# Supplementary material for: Synaptic dysfunction induced by glycine‐alanine dipeptides in C9orf72‐ALS/FTD is rescued by SV2 replenishment
Source: EMBO Mol Med. 2020 Apr 29;12(5):e10722. doi: 10.15252/emmm.201910722 (PMC7207170; doi:10.15252/emmm.201910722)
Supplement: Supplementary file 1 — Appendix [file EMMM-12-e10722-s001.pdf]

APPENDIX TABLES S1 - S2

APPENDIX TABLE S1
Exact *p*-values for Figures

Figure	Statistical Test	Comparison	*p*-value
2C	one-way ANOVA	GA length dependence cortical	0.0035
2D	one-way ANOVA	GA length dependence motor	<0.0001
3B	Cox proportional hazard	GA50 compared with control cortical	<0.0001
	log-rank test	GA50 compared with control cortical	<0.0001
	Cox proportional hazard	GA100 compared with control cortical	0.0025
	log-rank p value	GA100 compared with control cortical	<0.0001
3C	Cox proportional hazard	GA50 compared with control motor	0.0018
	log-rank test	GA50 compared with control motor	<0.0001
	Cox proportional hazard	GA100 compared with control motor	<0.0001
	log-rank p value	GA100 compared with control motor	<0.0001
4C	Sidak multiple comparison	GA50 versus GFP	<0.0001
5B	Uncorrected Fisher's LSD test	mCherry versus GA50 cortical	0.0009
	Uncorrected Fisher's LSD test	mCherry no calcium versus GA50 no calcium cortical	0.935
5C	Uncorrected Fisher's LSD test	mCherry versus GA50 motor	0.0266
	Uncorrected Fisher's LSD test	mCherry no calcium versus GA50 no calcium motor	0.9314
6C	one-way ANOVA	SV2 puncta GA length dependence compared with GFP cortical	<0.0001
	one-way ANOVA	Total neurite length GA length dependence compared with GFP cortical	0.6015
6D	one-way ANOVA	SV2 puncta GA length dependence compared with GFP motor	<0.0001
	one-way ANOVA	Total neurite length GA length dependence compared with GFP motor	0.4707
7C	unpaired t-test	isogenic versus c9 line	<0.0001
7D	unpaired t-test	isogenic versus c9 line	0.2789
7E	unpaired t-test	isogenic versus c9 line	0.0162
7F	unpaired t-test	isogenic versus c9 line	0.0269
7G	unpaired t-test	control versus c9 lines	0.1116
7H	unpaired t-test	control versus c9 lines	0.2606
8A	Sidak multiple comparison	GFP versus GA50	<0.0001
	Sidak multiple comparison	GFP versus GFP+ SV2	0.6237
	Sidak multiple comparison	GA50 versus GA50 +SV2	0.0037
	Sidak multiple comparison	GFP versus GA50+SV2	0.2859
8B	Uncorrected Fisher's LSD test	mCherry versus GA50	0.0248
	Uncorrected Fisher's LSD test	mCherry + SV2 versus GA50 + SV2	0.1834
8C	Cox proportional hazard	GA50 compared with control cortical	<0.0001
	log-rank test	GA50 compared with control cortical	<0.0001
	Cox proportional hazard	GA50 +SV2 compared with control cortical	0.0043
	log-rank p value	GA50 +SV2 compared with control cortical	<0.0001
8D	Cox proportional hazard	PR50 compared with control cortical	<0.0001
	log-rank test	PR50 compared with control cortical	<0.0001
	Cox proportional hazard	PR50 +SV2 compared with control cortical	<0.0001
	log-rank p value	P50 +SV2 compared with control cortical	<0.0001
8E	Cox proportional hazard	GA50 compared with control motor	0.0009
	log-rank test	GA50 compared with control motor	<0.0001
	Cox proportional hazard	GA50 +SV2 compared with SV2 control motor	0.0582
	log-rank p value	GA50 +SV2 compared with SV2 control motor	<0.0001
9A	unpaired t-test	wild type versus Tgx GA149	0.0495
9B	unpaired t-test	wild type versus Tgx GA149	0.8242
9C	unpaired t-test	wild type versus Tgx GA149	0.7246
10B	unpaired t-test	wild type versus Tgx GA149	0.0003
10C	unpaired t-test	wild type versus Tgx GA149	0.0873

APPENDIX TABLE S2
Exact *p*-values for Expanded View Figures

EV Figure	Statistical Test	Comparison	*p*-value
EV2C	one-way ANOVA	GFP versus GA length mitochondrial velocity cortical	0.9716
EV2D	one-way ANOVA	GFP versus GA length mitochondrial velocity motor	0.9742
EV2E	Dunnett's multiple comparison	GFP versus GA25	0.5159
	Dunnett's multiple comparison	GFP versus GA50	0.9645
	Dunnett's multiple comparison	GFP versus GA100	0.9672
	Dunnett's multiple comparison	GFP versus GA200	0.5146
	Dunnett's multiple comparison	GFP versus GA400	0.0296
EV2F	unpaired t-test	mCherry versus GA50, SV2 mRNA beacon cortical	0.5782
EV3A	one-way ANOVA	GA length dependence	0.1137
	Dunnett's multiple comparison	GFP versus GA25	0.0145
	Dunnett's multiple comparison	GFP versus GA50	0.0081
	Dunnett's multiple comparison	GFP versus GA100	0.3960
	Dunnett's multiple comparison	GFP versus GA200	0.0045
	Dunnett's multiple comparison	GFP versus GA400	0.9532
EV3B	one-way ANOVA	SynPhys puncta GA length dependence compared with GFP cortical	0.2725
	one-way ANOVA	PSD95 puncta GA length dependence compared with GFP cortical	0.9791
EV3C	one-way ANOVA	SynPhys puncta GA length dependence compared with GFP motor	0.6216
	one-way ANOVA	PSD95 puncta GA length dependence compared with GFP motor	0.9925
EV4C	unpaired t-test	control versus SV2 pRRL	0.0071
EV4D	unpaired t-test	control versus SV2 pRRL	0.0003
EV4F	Sidak multiple comparison	mCherry versus GA50	0.0002
		mCherry versus mCherry SV2 pRRL	0.0002
		mCherry versus GA50 SV2 pRRL	0.9846
		GA50 versus GA50 SV2 pRRL	0.0024
		mCherry SV2 pRRL versus GA50 SV2 pRRL	<0.0001
