# Supplementary material for: Synaptic dysfunction induced by glycine‐alanine dipeptides in C9orf72‐ALS/FTD is rescued by SV2 replenishment
Source: EMBO Mol Med. 2020 Apr 29;12(5):e10722. doi: 10.15252/emmm.201910722 (PMC7207170; doi:10.15252/emmm.201910722)
Supplement: Supplementary file 2 — Expanded View Figures PDF [file EMMM-12-e10722-s002.pdf]

Expanded View Figures

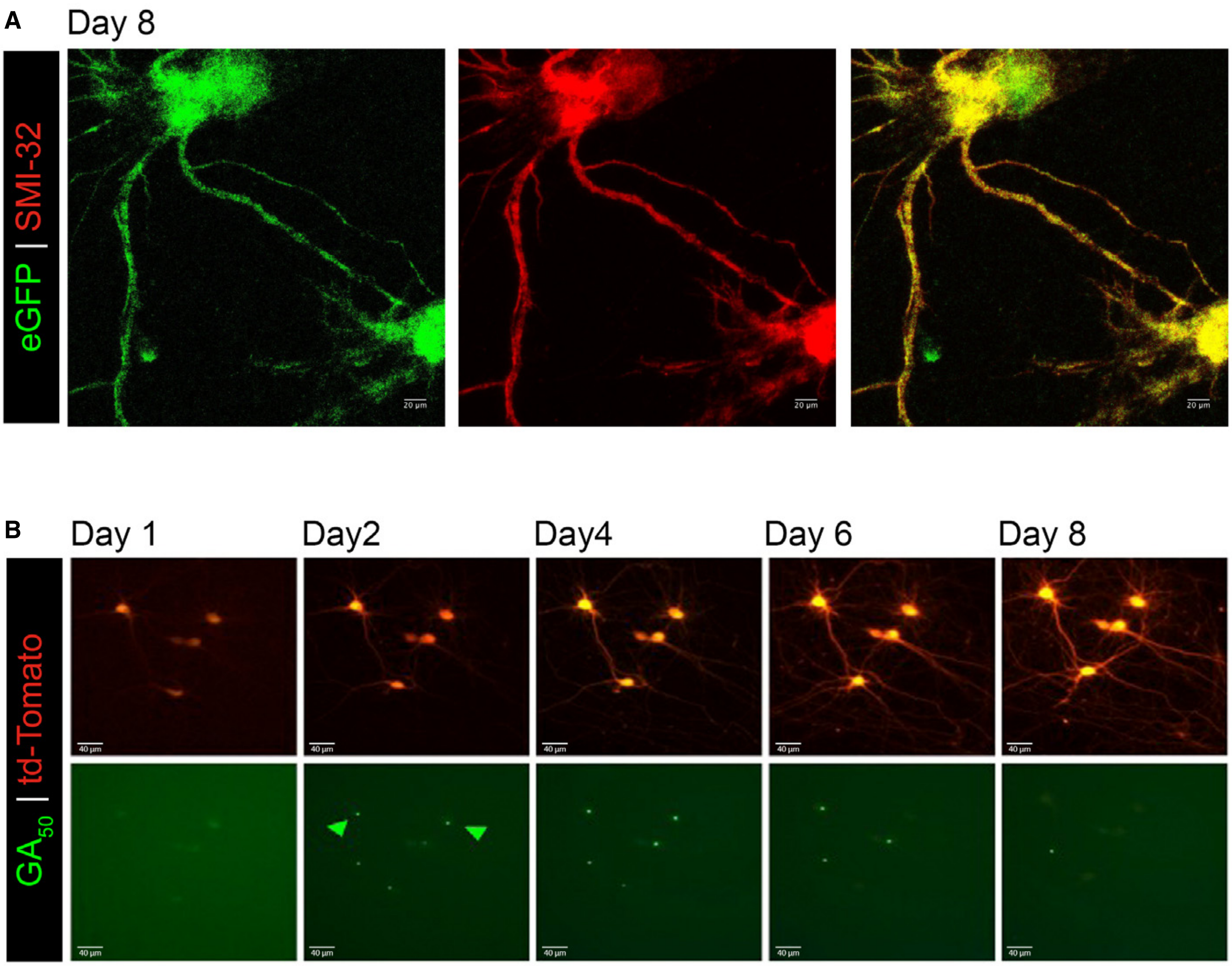

Figure EV1. GA aggregates are dynamic.

A eGFP was expressed in mature cortical neurons for 8 days and then immunostained for neurofilament (SMI-32, red) and eGFP (green). This representative z-stack confocal images demonstrate cellular viability and lack of GFP aggregation when expressed in the absence of GA_n dipeptides even at this extended time. 60× magnification, scale bar indicates 20 μm.

B Primary rat neurons were co-transfected with Td-tomato and eGFP-GA_{50} plasmid. The same neurons were imaged at 24-h intervals. Representative fields of td-Tomato (top) and eGFP-GA_{50} (bottom) co-positive cortical neurons at Days 1, 2, 4, 6, and 8 post-transfection follow individual cells over time. Highlighted with green arrows are GA aggregates that dissipate over the course of our imaging period, while the cells containing them remain viable. 20× magnification, scale bar indicates 40 μm.

© 2020 The Authors

© 2020 The Authors

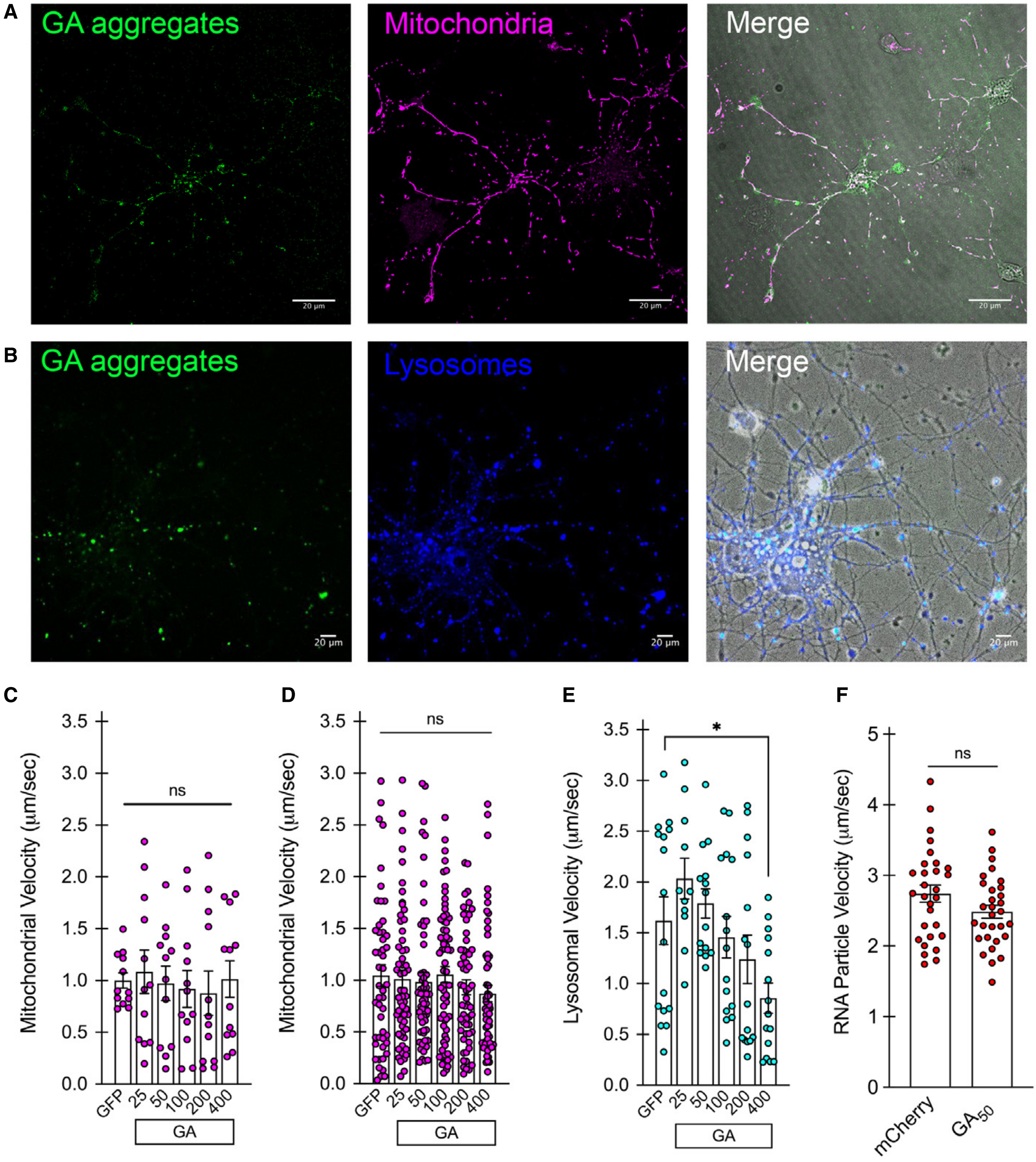

Figure EV2.

© 2020 The Authors

Figure EV2. Cellular trafficking is unaffected by the presence of neuritic GA aggregates.
Cortical and motor neurons underwent live-cell imaging after 48 h of eGFP-GA_n peptide expression to determine mobility of cargoes within neurites. MitoTracker Deep Red FM dye (50 nM)-labeled mitochondria, LysoTracker Deep Red (50 nM)-labeled lysosomes, SYTO RNASelect-labeled total cellular mRNA.

A Example of a cortical neuron expressing eGFP-GA_{50} (green) with labeled mitochondria (magenta). Right panel shows merge with the addition of brightfield overlay, 40× magnification, scale bar indicates 20 μm.

B Example of a cortical neuron-expressing eGFP-GA_{50} (green) with labeled lysosomes (blue). Right panel shows merge with the addition of brightfield overlay, 40× magnification, scale bar indicates 20 μm.

C, D Quantification of mitochondrial velocity within cortical neuron neurites (C) or motor neuron neurites (D) expressing eGFP-GA_n. Data presented as mean ± SEM. One-way ANOVA, *post hoc* Dunnett's multiple comparison test. Velocity measurements from 10 mitochondria were assessed from each of three biological replicates for each GA repeat length in each neuronal population.

E Quantification of lysosomal velocity within cortical neuron expressing eGFP-GA_n. Overall, GA dipeptides did not produce significant alterations in lysosomal mobility. Compared with control neurons expressing GFP, only GA_{400} induced a significant reduction in lysosomal trafficking velocity, $^*P < 0.05$. Data presented as mean ± SEM. One-way ANOVA, *post hoc* Dunnett's multiple comparison test. Velocity measurements from 10 lysosomes were assessed from each of three biological replicates for each GA repeat length.

F Quantification of total RNA velocity within cortical neuron neurites expressing mCherry-GA_{50} or mCherry alone. Data presented as mean ± SEM. Unpaired *t*-test. Velocity measurements from 10 mRNA puncta were assessed from each of three replicates for each condition.

Data information: Exact *P*-values can be found in Appendix Table S2.

© 2020 The Authors

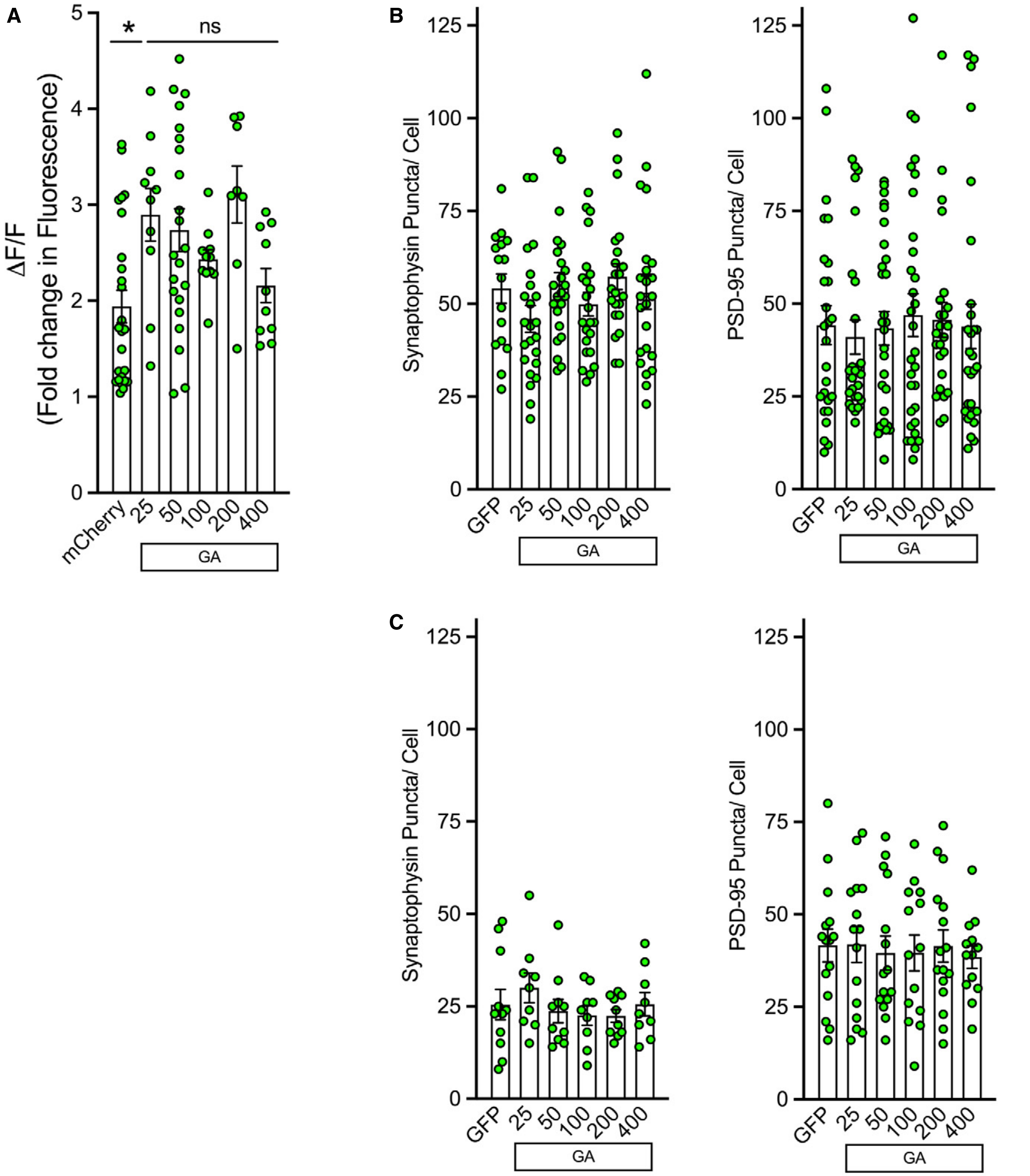

Figure EV3.

 © 2020 The Authors

Figure EV3. Synaptophysin and PSD-95 remain unaltered in neurons containing GA aggregates.

A Primary cortical neurons were co-transfected with mCherry or mCherry-GA_n and GCaMP6f. After 48 h, mCherry-positive cells were determined. Green fluorescence intensity was then recorded from identified neurons. Basal fluorescence was monitored prior to induced depolarization via perfusion with ACSF containing 50 mM KCl. Graphical representation is the quantification of peak change in fluorescence (ΔF) following ACSF perfusion normalized to basal fluorescence (F), ΔF/F. A significant increase was observed in mCherry-GA_n containing cells (GA_{25}, GA_{50}, GA_{200}) (*$P < 0.05$); however, a length-dependent association with Ca^{2+} influx levels was not detected. Data presented as mean ± SEM. One-way ANOVA, *post hoc* Dunnett's multiple comparison test. A total of 8–25 cells per condition were pooled from $n = 5$ independent biological replicates.

B, C eGFP-GA_n dipeptides were expressed in cortical or motor neurons for 48 h and then immunostained for neurofilament and either synaptophysin or PSD-95. Z-stack confocal images were captured at 60× magnification; puncta were quantified by ImageJ through manual counting. (B) Quantification of synaptophysin (left) or PSD-95 (right) puncta along neurites in eGFP-GA_n expressing cortical neurons compared with eGFP-only expressing cells revealed no significant differences. (C) Quantification of synaptophysin (left) or PSD-95 (right) puncta along neurites in eGFP-GA_n expressing motor neurons compared with eGFP-only expressing cells revealed no significant differences. Data presented as mean ± SEM. One-way ANOVA, *post hoc* Dunnett's multiple comparison test, 15–20 cells derived from $n = 3$ independent biological replicates.

Data information: Exact *P*-values can be found in Appendix Table S2.

Figure EV4. SV2 is upregulated following rSV2a-eGFP-pRRL lentiviral transduction.

A mCherry-GA_n dipeptides were expressed in cortical or motor neurons for 48 h and then immunostained for SV2 and a neuritic marker for neurofilament (SMI-32). Representative z-stack confocal images of neurites (SMI-32, cyan), showing colocalization of mCherry-GA_{50} aggregates (red) with SV2 puncta (green). Arrows highlight colocalized areas; scale bars indicate 10 μm.

B A custom beacon probe was generated against SV2 mRNA, which was co-transfected along with eGFP or eGFP-GA_{50} constructs and the cell-filling td-Tomato plasmid into mature cortical neurons. Shown is a representative image of this mRNA beacon (blue) localized to td-Tomato positive neurites (red). Arrows highlight colocalized areas; scale bar indicates 5 μm.

C mRNA was Trizol extracted from three independent wells of rSV2a-eGFP-pRRL lentiviral-transduced wells of cortical neurons and non-transduced controls following 4 days of transduction. RNA was converted to cDNA using the Superscript First-strand kit, and qPCR was performed using PowerUp SYBR Green. Measurements were normalized to the housekeeping gene GAPDH and then to non-transduced controls. Analysis was performed using the ΔΔCT method. $2^{\Delta\Delta CT}$ ± SE is presented. Quantification revealed a 1.99-fold increase in SV2 mRNA. Unpaired *t*-test, **$P < 0.01$, $n = 3$ biological replicates.

D Whole-cell lysates were generated from three independent wells of rSV2a-eGFP-pRRL lentiviral-transduced wells of cortical neurons and non-transduced controls following 4 days of transduction. Lysates were immunoblotted for SV2 and normalized to total protein loading. Quantification band intensities revealed a significant upregulation of SV2 protein levels in rSV2a-eGFP-pRRL-transduced neurons. Data presented as mean ± SEM. Unpaired *t*-test, ***$P < 0.001$, $n = 3$ independent biological replicates.

E, F eGFP-GA_{50} dipeptides were expressed in primary cortical neurons for 48 h with or without the addition of SV2 pRRL lentivirus. Cells were immunostained for neurofilament (gray) and SV2 (red). (E) Representative z-stack confocal images of neurites from mCherry, mCherry-GA_{50}, mCherry-GA_{50}, +SV2 pRRL, and mCherry + SV2 pRRL-expressing cortical neurons. 60× magnification, scale bar indicates 5 μm. (F) Quantification of SV2 puncta along neurites by ImageJ manual counting. The significant reduction in SV2 puncta upon GA_{50} expression was significantly rescued to normal levels by exogenous expression through transduction with the SV2 pRRL lentiviral vector. Data presented as mean ± SEM. One-way ANOVA, *post hoc* Dunnett's multiple comparison test, **$P < 0.05$, ***$P < 0.001$, ****$P < 0.0001$, $m = 5$ neurons per condition from $n = 3$ independent biological replicates.

Data information: All exact *P*-values can be found in Appendix Table S2.
Source data are available online for this figure.

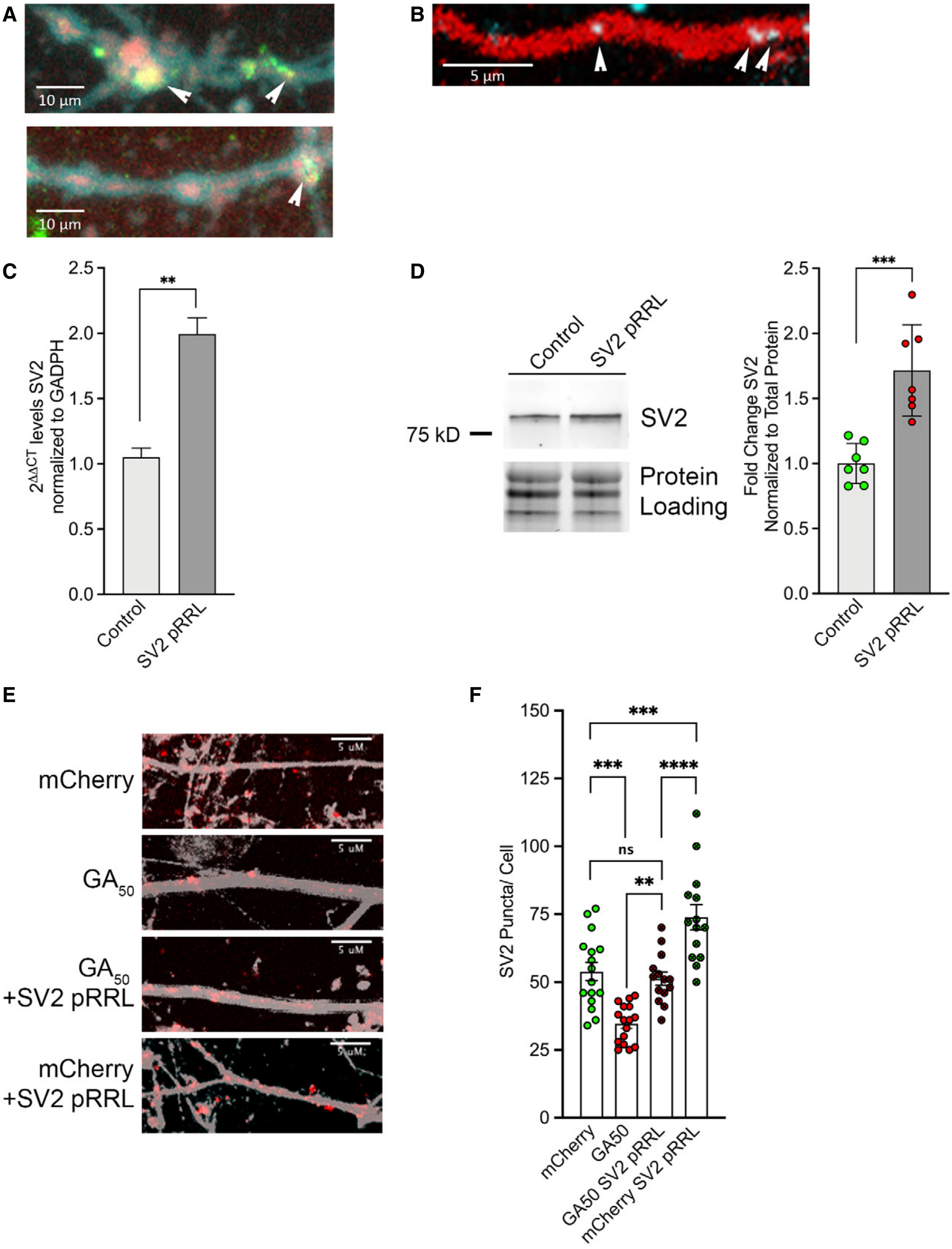

Figure EV4.

 © 2020 The Authors
