# Supplementary figures and images for: Synaptic dysfunction induced by glycine‐alanine dipeptides in C9orf72‐ALS/FTD is rescued by SV2 replenishment
Source: EMBO Mol Med. 2020 Apr 29;12(5):e10722. doi: 10.15252/emmm.201910722 (PMC7207170; doi:10.15252/emmm.201910722)

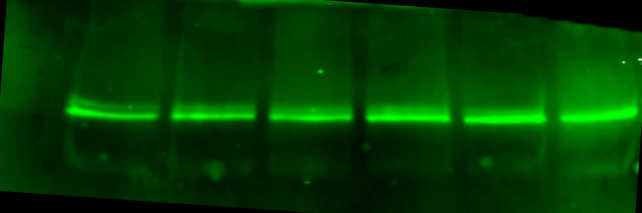

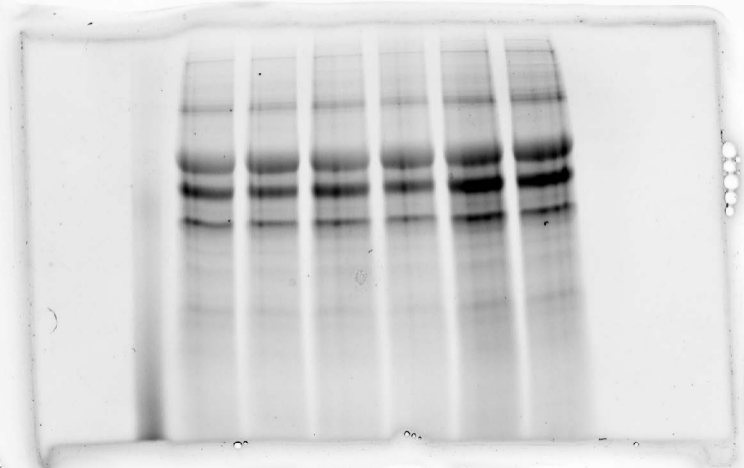

Supplement: Supplementary file 3 — Source Data for Expanded View [file EMMM-12-e10722-s005.pdf]

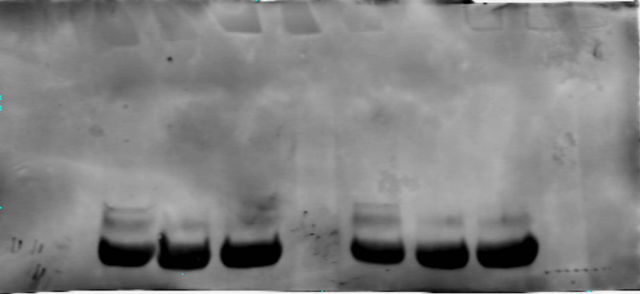

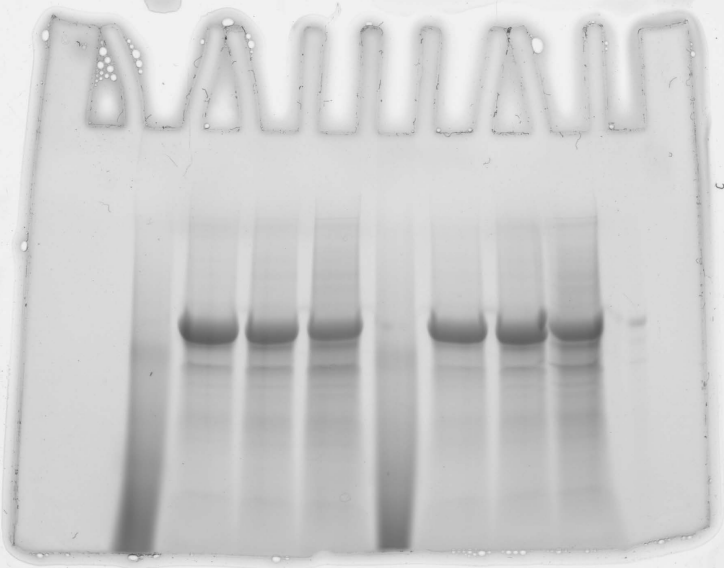





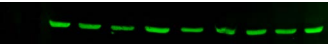

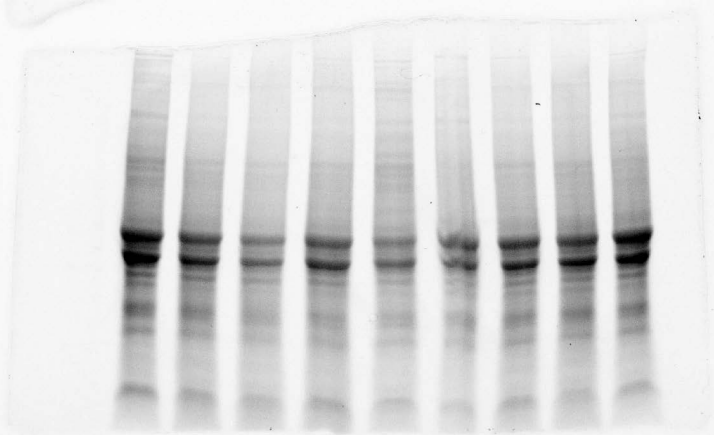

Supplement: Supplementary file 5 — Source Data for Figure 7 [file EMMM-12-e10722-s003.pdf]

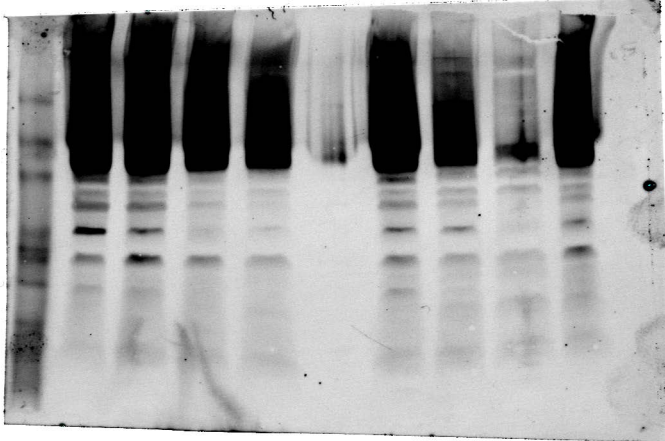

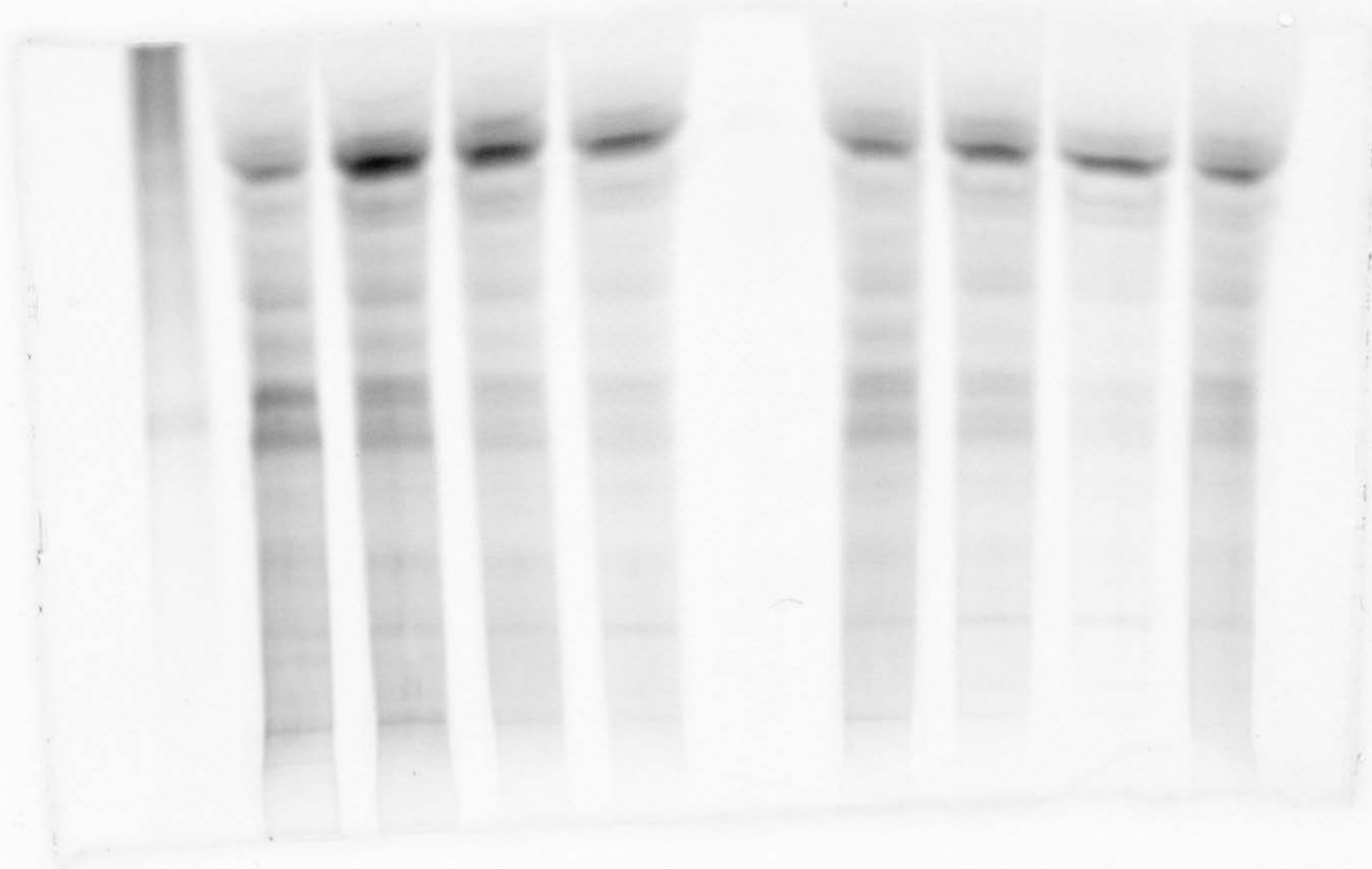

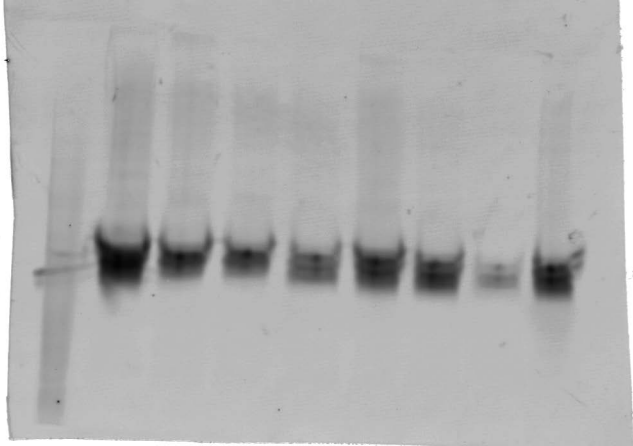



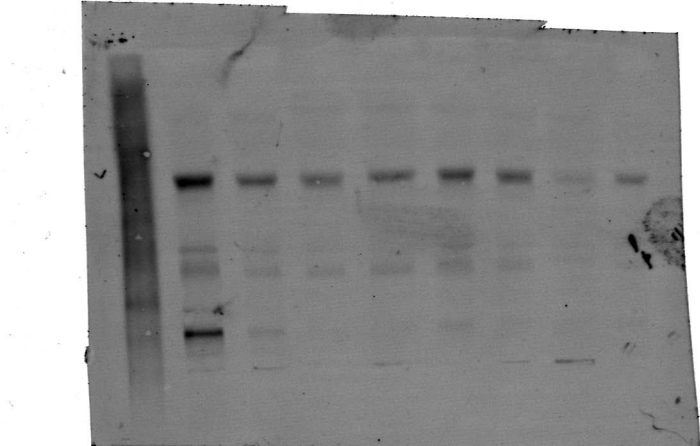

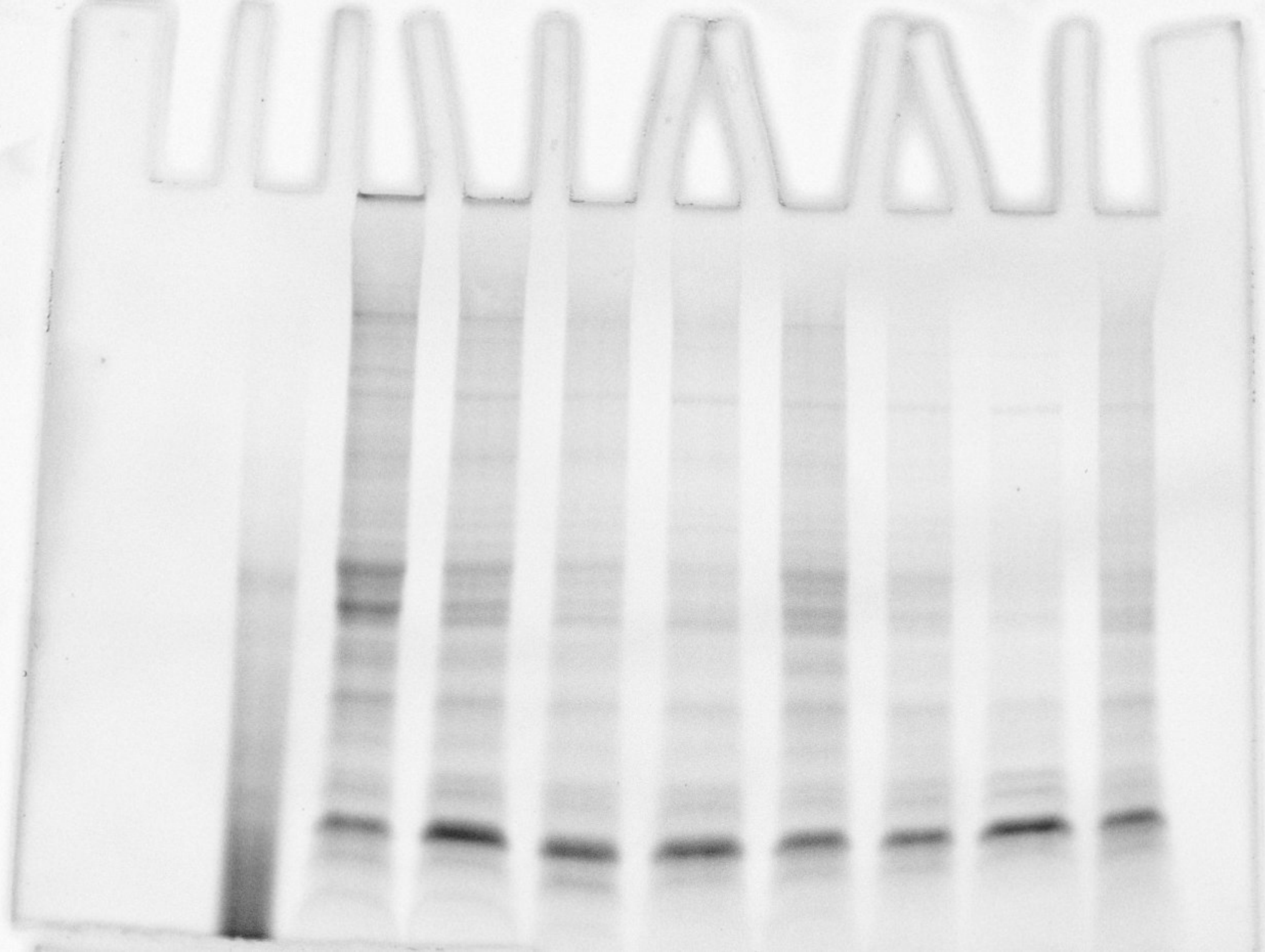

Supplement: Supplementary file 6 — Source Data for Figure 9 [file EMMM-12-e10722-s004.pdf]
